# Ethical Issues in mHealth Research Involving Persons Living with HIV/AIDS and Substance Abuse

**DOI:** 10.1155/2013/189645

**Published:** 2013-09-19

**Authors:** Alain B. Labrique, Gregory D. Kirk, Ryan P. Westergaard, Maria W. Merritt

**Affiliations:** ^1^Johns Hopkins Bloomberg School of Public Health, 615 North Wolfe Street, Baltimore, MD 21205, USA; ^2^University of Wisconsin-Madison, 1685 Highland Avenue MFCB 5220, Madison, WI 53705-2281, USA; ^3^Johns Hopkins Berman Institute of Bioethics, 1809 Ashland Avenue, Baltimore, MD 21205, USA

## Abstract

We aim to raise awareness and stimulate dialogue among investigators and research ethics committees regarding ethical issues that arise specifically in the design and conduct of mHealth research involving persons living with HIV/AIDS and substance abuse. Following a brief background discussion of mHealth research in general, we offer a case example to illustrate the characteristics of mHealth research involving people living with HIV/AIDS and substance abuse. With reference to a well-established systematic general ethical framework for biomedical research with human participants, we identify a range of ethical issues that have particular salience for the protection of participants in mHealth research on HIV/AIDS and substance abuse.

## 1. Introduction

 In the past decade, mobile phone technology has become nearly ubiquitous in most developed country contexts, crossing socioeconomic boundaries and in some populations displacing traditional landline infrastructure. Similar trends have been noted globally, as current estimates suggest that nearly 6.1 billion mobile subscriptions exist in 2013 [1]. The pervasive growth of this technology has resulted in innovations across sectors of agriculture, education, and even health, focused around a new domain of research and implementation science termed “mHealth” or mobile health. Classical medical information systems and technologies have, for decades, been centered on the highly tethered, facility-based patient record, and other management systems. The advent of mHealth has led both researchers and patients to explore the potential for mobile technologies to improve health outcomes and lower costs by increasing patient engagement, improving provider quality, and optimizing efficiency in health care. mHealth opens new avenues for research insight, as this ubiquitous technology allows much more frequent data collection about participants' behavior, location, and physiology, sometimes in real time [2]. 

 In the past 5 years, a growing body of mHealth research has emerged, exploring the role of these technologies in improving preventive and curative care. In HIV, a number of research projects have explored how mobile phones can be used to improve adherence to antiretroviral treatment in low-resource settings [3, 4] to provide decision support to frontline health workers [5, 6] and to introduce the benefits of continuous care in places where this was previously impossible. mHealth strategies have been used to improve patient care and self-efficacy by improving adherence to complex antiretroviral regimens, reducing missed appointments, and connecting individuals to care when and where they need it. Governments and program agencies have used mHealth approaches to mobilize awareness of HIV prevention and treatment, promote testing, and advocate for support for persons living with HIV/AIDS [7–9]. 

Still more sophisticated wearable mobile devices (e.g., accelerometers to measure physical activity and sensors to measure heart rate, blood pressure, or other biological processes) can harness the capacity of built-in geographic sensing and the ability to connect to other wireless devices. Such systems enable health programs and research studies to define profiles of behavior and risk exposure with more granularity than ever before in real time. Noninvasive sensors allow an individual's physiology to be monitored continuously, with little engagement by that individual, while text message or app-driven prompts can inquire about behaviors, mood, or even ideation frequently throughout the day. This emergent space, described as *ecologic momentary assessment* (EMA), offers exciting epidemiologic potential, while introducing new ethical concerns and caveats.

## 2. Case Example

In order to set the stage for our discussion of ethical issues, we present a case example illustrating typical characteristics of mHealth research involving persons living with HIV/AIDS and substance abuse. The example is based on an ongoing study led by 2 of the authors (RPW and GDK) at Johns Hopkins University.

*Trial of Technology-Enhanced Peer Health Navigation.* Investigators are following a cohort of injection drug users (IDUs) who are living with HIV. This pilot study aims to test the feasibility and acceptance of an intervention featuring peer health navigation in combination with a smartphone application to improve medication adherence and attendance at clinic appointments. Previous research with this population has shown that patients who inject drugs are often engaged in HIV care sporadically, and HIV viral suppression resulting from antiretroviral therapy may be short-lived. HIV treatment is often interrupted by relapses into drug use, incarceration, and other psychosocial stressors. The investigators hypothesize that individualized psychosocial support (including assistance with overcoming logistical barriers to care) provided by peer health navigators will improve the likelihood that IDUs living with HIV will remain engaged in HIV care over 12 months of followup. They further hypothesize that because peer health navigation is time-and resource-intensive, incorporating an mHealth application into the intervention will improve its efficiency and scalability. The research team has developed a customizable smartphone application that facilitates communication among patients and support staff and collects real-time data describing common risk factors for nonadherence such as negative-mood states and drug and alcohol use.

The study is recruiting people living with HIV/AIDS who have a history of problematic drug or alcohol use and who are not consistently engaged in care. The trial design specifies that participants will be randomly assigned to usual care (HIV-oriented primary care with clinic-based medical case management) or the technology-enhanced peer navigation intervention. Participants in the intervention arm will be assigned a peer health navigator and will be given a smartphone running the study application. They will be expected to carry the phone at all times and may use it for personal calls, web applications, or to contact their peer navigator or clinic nurse as needed. The application will also prompt participants to respond to brief questionnaires 1-2 times daily that ascertain the level of stress they are experiencing, drug use or cravings, and anticipated barriers to keeping clinic appointments or adhering to their prescribed antiretroviral regimen. The data will be reviewed daily by research staff and the peer navigators, who will initiate contact with participants whose responses indicate they may be at high risk of disengaging from care. Participants in the usual care and intervention groups will be compared over 1-year followup with regard to missed appointments and to achieving and sustaining viral suppression in response to antiretroviral therapy. 

## 3. A Framework to Address Salient Ethical Issues

In order to articulate the salient ethical issues raised by this example, we organize our discussion with reference to the systematic general ethical framework for biomedical research developed by Emanuel and colleagues and now well-established in the literature [10]. The framework, a critically reflective synthesis and elaboration of the most important existing ethical guidelines, presents 8 principles as necessary for the ethical justification of biomedical research with human participants: collaborative partnership, social value, scientific validity, fair participant selection, favorable risk-benefit ratio, independent review, informed consent, and respect for participants. For each principle, the framework specifies several benchmarks meant to indicate what its fulfillment requires in practice. All the principles are generally applicable to any sort of biomedical research with human participants. In what follows, we highlight three principles (and related benchmarks) as salient to the use of mHealth technology in research involving persons living with HIV/AIDS and substance abuse: scientific validity, fair participant selection, and favorable risk-benefit ratio. These are the principles that appeared most salient to us based on our experience of conducting mHealth research (ABL; GDK; RPW) and performing ethical review of mHealth research protocols (MWM). Depending on one's background and experience, other principles may also assume prominence, and we recommend to interested readers the exercise of applying the Emanuel et al. framework in full to their own research.

### 3.1. Scientific Validity

In order to justify the exposure of human participants to the burdens and risks of biomedical research, the research must be designed and conducted so as to produce scientifically valid results that are “interpretable and useful in the context of the health problem” [11]. In our case example, the eventually intended beneficiaries are IDUs living with HIV who are served by health systems relevantly similar to the one under study. The hope is that some form of smartphone “patient support” application will enhance the population-level impact (efficiency and scalability) of an otherwise resource-intensive peer health navigation intervention. Eventually, it is hoped that individual IDUs living with HIV could use such technology to benefit from antiretroviral therapy and other aspects of HIV care to a significantly greater degree than they would otherwise have been able to do in the absence of the technology. Data to be generated by the study, then, should be capable of interpretation and use in the context of the behaviors and risk factors that place members of the intended beneficiary population at high risk of treatment failure.

The application of this point to mHealth technologies raises the ethical issue of responsibility for doing high-quality formative research. By the time any mHealth technology reaches the stage of being studied in the context of biomedical research, it should have gone through a foundational design process that promotes simplicity and ease of use, thereby minimizing its burdens for intended users. Formative research that engages with end users is a key part of the design process, increasingly recognized as a necessary process component, or “best practice” in mHealth design. End users should be engaged not only in the interface design but also in pre-testing and in providing feedback on whether a technology is excessively cumbersome or burdensome. To ensure that the data generated and communicated through mHealth research are useful and interpretable to end users, information access portals need to be designed for navigability, quality, presentation, and accuracy. The study protocol described in the case example resulted from several years of interaction between the study investigators and target participant groups through performance of a series of iterative field trials using similar methodologies. The investigators conducted formal assessments of feasibility and participant acceptability, which informed the design and implementation of the subsequent research. 

Once an application of mHealth technology has gone through the design process and is suitable for study in the context of biomedical research, there should be a clear scientific justification for all data elements being collected. As detailed below in the discussion of risks, mHealth data collection may introduce or increase risks of various harms including social marginalization, psychological stress, invasion of privacy, or breach of confidentiality. For each variable on which data are collected and for each of the proposed interactions with the participant, there should be an a priori hypothesis justifying its inclusion; for example, that the data collected will improve clinical insight, the engagement strategy will improve adherence, or the patient feedback loops will increase quality of care. For example, geolocation data should not be collected merely as part of metadata for future data mining; if they are to be collected, relevant justifying hypotheses are required. 

Additional care is required to take into consideration disparities in socioeconomic status and life circumstances between technical designers and end users. In the case example, formative research suggested that a minority of IDUs participating in the cohort study had used a smartphone. Conversely, smartphone ownership is widespread in the social and professional networks of investigators. Such differences in experience with technology may lead to underappreciation of the challenges likely encountered by participants in the study and may threaten the validity of the desired data describing feasibility and acceptance of the intervention. Among relevant burdens is the cost associated with owning and operating mobile devices over time. While investigators often provide the necessary devices to participants during the course of a research study, failure to acknowledge the financial burden associated with using the technology outside of the research setting may threaten the sustainability and, ultimately, the real-world impact of mHealth interventions. Similar ethical challenges are often faced when conducting research in low-resource settings in the developing world, where socioeconomic disparities between research teams and participants tend to be pronounced. 

### 3.2. Fair Participant Selection

Benchmarks of fair participant selection include several requirements [11]. The selection of research populations must be justified in terms of the scientific validity and social value of the research (i.e., eventual generalizable knowledge leading to improvements to health or health care for the intended beneficiary population). Inclusion and exclusion criteria for individual participants must be similarly justified. If the inclusion of vulnerable populations and individuals is necessary on grounds of scientific validity and social value, additional protective safeguards must be in place. 

In our case example, the selection of the research population, IDUs who are living with HIV, appears readily justifiable. Given that the development of interventions to support persons living with HIV/AIDS and substance abuse may justifiably require including IDUs in biomedical research, it is important to consider vulnerability. Participating populations and individuals may be vulnerable due to the stigmatization of HIV/AIDS or economic deprivation and may often be exposed to elevated risk of incarceration from engagement in criminalized behaviors. These vulnerabilities make it imperative, in general, to include relevant safeguards for the protection of research participants living with HIV/AIDS and substance abuse, as detailed below in the discussion of risks. In addition, research on mHealth interventions in particular might exacerbate preexisting vulnerabilities. In recruiting participants, it is inappropriate for investigators to target intentionally and specifically those who, due to low income or unstable housing, may not have access to newer devices and other modes of mobile technology and may thereby be unduly influenced by the incentive of access to technology in a way that more affluent groups or individuals would not be. 

A further ethical issue specific to mHealth arises regarding the inclusion and exclusion of individual participants. A de facto inclusion criterion for participation in mHealth research involving interactive data collection, even if it is not formally specified in the research protocol, is some degree of fluency in the use of mobile digital technology: for example, being able to send an SMS or being familiar with smartphone operations. Some older or less educated prospective participants—indeed, perhaps those most in need of supplemental patient support—are thereby excluded. Strategies to overcome such barriers to participation may be warranted, perhaps in the form of short “trainings” to impart the necessary skills to perform basic technical functions. Again, in many low-resource settings, both in developing countries and among underprivileged populations within developed countries, it is possible to overcome such technical barriers through the use of pictorial menus or simple icon-driven interactions; for instance, text-based queries can be replaced by recorded voice messages. These, of course, come with additional costs to the researcher but may prevent unnecessary exclusion of the least-advantaged members of the target population.

### 3.3. Favorable Risk-Benefit Ratio

The principle of favorable risk-benefit ratio requires that risks to individual research participants be delineated, justified, and minimized [10]. Research with persons living with HIV/AIDS and substance abuse, while sometimes offering individual participants the prospect of direct benefit (such as through clinically relevant test results and referral to needed clinical followup), at the same time requires special care in delineating and minimizing research-related risks such as invasion of privacy or breach of confidentiality, since both HIV/AIDS and substance abuse carry some social stigma and substance abuse may involve criminalized behaviors. We focus here on the ways in which mHealth research, specifically, might exacerbate preexisting risks or introduce new risks for persons living with HIV/AIDS and substance abuse. 

#### 3.3.1. Physical, Social, Behavioral, and Psychological Risks

Investigators should think through the following concerns, ensuring that they take into account the life circumstances of the groups, communities, or populations from which they seek to enroll research participants. The provision of a high-value mobile mHealth device might expose participants to physical targeting for theft if the technology is far beyond what is “normal” among their peers; or it might enable high-risk behavior through exchange (or resale) of the device for money or drugs; or it might induce psychological stress due to perceived responsibilities of ownership or safeguarding. Researchers can address these sorts of concerns by developing studies that utilize the participants' own phones or devices or by emphasizing technologies currently accessible to their peer group. The market value of the devices used in mHealth research can be minimized by restricting nonstudy features and by incorporating technology that allows the remote inactivation of the device as a deterrent from diverting or attempting to resell the device. Another behavioral concern is that some participants might perceive mHealth systems as a substitute for standard care (as when algorithm-based “personal feedback” is mistaken for live monitoring). A related risk is the creation of a false belief on the part of participants that mobile monitoring in itself offers additional protection for high-risk behaviors. A recent study in Uganda by Jamison and colleagues found the unexpected result that providing mobile-phone-based information about sexual health actually increased levels of promiscuity among users—another possible unintentional consequence of access to information that changes behaviors in ways unforeseen by the investigators [12]. Researchers could try to address this type of concern through a combination of counseling (both during the informed consent process and as the study progresses) and safety monitoring based upon ongoing data collection.

Studies using biosensors need to guard against social risks of further marginalization and psychological risks due to the perception of looking “different” by allowing for appropriate concealment of sensors. Advances in sensor miniaturization allow for complex biosensors to be concealed in unobtrusive formats as benign as a large “Band-Aid.” Wireless technologies such as Bluetooth (TM) allow for data to be transmitted between sensors and mobile phones without obvious wires or leads, while advances in battery life and low-power circuit designs permit extended device use without requiring participants to frequently recharge their mHealth devices.

#### 3.3.2. Risks to Privacy and Confidentiality

Given that mobile digital data exchange is a defining attribute of mHealth, risks to privacy and confidentiality are highly salient in mHealth research. While both privacy and confidentiality must be protected, adequate protection of both requires noting the distinction between the two: “Privacy can be defined in terms of having control over the extent, timing, and circumstances of sharing oneself (physically, behaviorally, or intellectually) with others. Confidentiality pertains to the treatment of information that an individual has disclosed in a relationship of trust and with the expectation that it will not be divulged to others in ways that are inconsistent with the understanding of the original disclosure without permission” [13]. 

Ecologic momentary assessment (EMA), as described above, is potentially invasive to privacy, as it can continuously or intermittently record and transmit detailed information about where a person is and, to some extent, what they are doing. Physiologic EMA poses risks of inadvertent insight into a participant's behavior (e.g., through activity patterns or respiratory signatures), revealing information beyond the profiles that are scientifically justified and being sought through data collection. Such potential violations of privacy accompanying EMA pose distinct problems related to informed consent, as privacy might turn out to be violated in ways that were not anticipated *ex ante *by either investigators or participants. Workarounds to minimize intrusiveness include the use of frequent electronic permission prompts or reminders that monitoring is on or off, and the possibility of setting limits to the hours during which data will be collected (e.g., 9 a.m. to 9 p.m.) so as to avoid infringement on “personal” time.

The fate of text (SMS) messages is inherently uncontrolled as messages can be read by persons other than the intended recipient of the information; moreover, messages can be forwarded and can remain resident on unsecured devices for the lifetime of the technology. Text messages containing reminders to take medications, for example, could result in unintended disclosure of the presence of a medical condition even without specifying any details of the type of treatment. The onus is on researchers to protect identifiable data and to ensure that participant confidentiality is maintained. In some instances where sophisticated systems for data storage, encryption, and authentication are not available, code words or euphemistic coded messages have been used in order to guard against the inadvertent disclosure of private information to third-party bystanders. Some institutions, beyond complying with legal requirements such as the United States Health Insurance Portability and Accountability Act (HIPAA), have implemented policies that limit electronic communication to patients for clinical care. While it may be possible to bypass such restrictions in the context of research, they may impede implementation and scale-up of beneficial interventions into clinical settings. Consultation with local institutions that provide care to the target population when developing an mHealth research protocol is therefore important to ensure that the intervention appropriately addresses the needs and limitations of all relevant stakeholders. 

Regarding the confidentiality of research data, it is of special note that the very behaviors and risk factors that place substance users at high risk of treatment failure are also ones that expose them to legal risk. Accordingly, the protection of confidentiality in mHealth research studies that collect data on these behaviors and risk factors requires extra care above and beyond standard measures, including consideration of obtaining a Certificate of Confidentiality, a legal tool available in the United States [14], or a similar legal safeguard if available in other countries. For mHealth generally, data security issues are a major source of regulatory concern, from transmission of data to local storage of data, and “ownership” of what is otherwise considered confidential patient data. In late June of 2013, the Thomson Reuters Foundation, in collaboration with the mHealth Alliance and other partners, released a report entitled “Patient privacy in a mobile world,” reviewing the state of mHealth security guidelines and directives globally [15]. In addition, the International Organization for Standardization (ISO) has issued health information management guidelines that provide recommendations on appropriate safeguards of patient data, relevant to mHealth research and implementations [16, 17]. 

## 4. Conclusion

The advent of mHealth technologies has extended, in ways previously unimaginable, our ability as researchers to study, track, and understand high-risk behaviors within the individual and geospatial contexts in which they occur. This unprecedented availability of granular, real-time data may produce novel strategies that improve patient outcomes and increase self-efficacy. However, the rapid rate of adoption of these methods and technologies requires careful consideration of the ethical issues associated with their use. Existing standards and best practices may need to be supplemented with new guidelines to ensure that patients and vulnerable populations are appropriately protected. The pace of technological innovation sometimes exceeds that of ethical standards and guidance. We hope that this discussion will serve as a springboard for continued conversation to minimize this gap moving forward, providing mHealth researchers and implementers a starting point and a framework to examine and mitigate potential risks associated with their work on an important frontier of public health innovation.
